# Minimal Clinically Important Difference of Average Daily Steps Measured Through a Consumer Smartwatch in People With Mild-to-Moderate Parkinson Disease: Cross-Sectional Study

**DOI:** 10.2196/64213

**Published:** 2025-07-29

**Authors:** Edoardo Bianchini, Marika Alborghetti, Silvia Galli, Clint Hansen, Alessandro Zampogna, Antonio Suppa, Marco Salvetti, Francesco Ernesto Pontieri, Domiziana Rinaldi, Nicolas Vuillerme

**Affiliations:** 1 Department of Neuroscience, Mental Health and Sensory Organs (NESMOS), Sapienza University of Rome, Via di Grottarossa 1035, Rome, 00189, Italy, 39 0633775579; 2 AGEIS, Université Grenoble Alpes, Grenoble, France; 3 Department of Human Neurosciences, Sapienza University of Rome, Rome, Italy; 4 Department of Neurology, Kiel University, Kiel, Germany; 5 IRCCS Neuromed Institute, Pozzilli, Italy; 6 Institut Universitaire de France, Paris, France

**Keywords:** gait, Parkinson’s disease, sensors, step count, smartwatch, MCID, wearables, activity monitor, motor symptoms, digital mobility outcomes, digital health, cross sectional study, linear regression, effectiveness, minimal clinically important difference

## Abstract

**Background:**

Recent studies demonstrated the validity, reliability, and accuracy of consumer smartwatches for measuring daily steps in people with Parkinson disease (PD). However, no study to date has estimated the minimal clinically important difference (MCID) for average daily steps (avDS), measured through a consumer smartwatch in people with PD.

**Objective:**

This study aimed to calculate the MCID of avDS, measured through a commercial smartwatch (Garmin Vivosmart 4) in people with PD.

**Methods:**

People with PD with a disease stage <4, without cognitive impairment, and who were able to walk unaided, wore a Garmin Vivosmart 4 smartwatch for 5 consecutive days on the wrist least affected by the disease, allowing the computation of avDS. To define the 3 levels of MCID for avDS, we used an anchor-based method linked to: (1) scales capturing subtle changes in global mobility and motor functions, (2) clinical and health-related measures, and (3) disease-related patient-reported outcomes. Linear regressions, Student *t* test, and ANOVA were used to estimate the minimal change in avDS based on anchors relevant change. For each level, the overall MCID was calculated as the average of the variables included, and the range was reported.

**Results:**

A total of 100 people with PD were enrolled. Participants took on average 5949 (SD 3034) daily steps, ranging from 357 to 12,620. The MCID of avDS anchored to standardized measures of motor symptoms and mobility was 581 steps/day (range  554‐608) or around 10% of mean avDS in our population. The MCID of avDS anchored to clinical and health-related variables was 1200 steps/day (range  350‐1683), or around 20% of mean avDS in our population. Finally, the MCID of avDS anchored to disease-related patient-reported outcomes was 1592 steps/day (range 594‐2589), or around 27% of the mean avDS in our population.

**Conclusions:**

These findings could be relevant for designing future clinical trials involving avDS as a digital mobility outcome in daily life for people with PD and evaluating the effectiveness of the intervention promoting free-living walking in this population.

## Introduction

Parkinson disease (PD) is a neurodegenerative disease characterized by both motor and nonmotor symptoms. Among the motor manifestations of PD, gait disturbances are notably prevalent and encompass a variety of issues, such as a shuffling gait, reduced step length and diminished automaticity, decreased arm swing, increased double support time, altered cadence, and periodic episodes such as freezing of gait (FOG) [1]. Walking, a fundamental daily activity, is crucial for maintaining functional independence and social well-being [2]. Gait disturbances, therefore, represent a particularly disabling category of symptoms in PD, significantly impacting the autonomy and quality of life of patients [3].

Gait characteristics can be examined through various methods, both in controlled and ecological settings. Recently, the use of wearable inertial sensors for gait analysis has gained considerable traction [4-6]. Specifically, the daily step count serves as an indirect yet rather easily obtainable indicator of walking and physical activity [7]. While this metric does not provide detailed gait analysis, there is established evidence linking a reduced daily step count to a range of health issues, including oncological and cardiovascular diseases [8], dementia [9], as well as an elevated risk of overall mortality [10-13]. In the context of PD, studies have consistently demonstrated a correlation between a decrease in daily steps and increased disease severity [14] as well as with levels of physical activity [15,16]. These studies have also proposed a minimum daily step goal of around 4200 to align with physical activity recommendations in the early stages of PD [17].

Step measurement can be accomplished using either research-grade or consumer devices. Research-grade devices are specifically developed for use by professionals in research or clinical settings and are generally recognized for their superior accuracy and reliability [18-21]. Despite this, they come with drawbacks such as higher costs, limited market availability, the need for specialized expertise for operation, and less focus on aesthetic appeal and user-friendliness. In contrast, consumer wearable devices are designed for the general public. These devices are easily accessible in the market and user-friendly, requiring no specialized knowledge for operation. However, they typically lack validation for research purposes and often do not include sophisticated algorithms or analysis software [22]. Despite this, there is an increasing focus in the literature on using consumer wearables for data collection and monitoring in neurological patients [23-25]. Both device types demonstrate reliable step counting in individuals with normal gait patterns [26], though research-grade devices show higher reliability across a wider range of conditions [27]. However, for individuals with nonphysiological gait, such as those with reduced walking speeds or those using walking aids, the performance of consumer wearables tends to diminish [18,28-31].

PD poses a unique challenge for gait analysis, as its associated disturbed gait patterns could adversely impact the reliability of device algorithms. Furthermore, the occurrence of tremors and dyskinesias in patients with PD may introduce additional background noise in recordings, potentially resulting in an overestimation of step counts [31,32]. Conversely, the presence of rigidity and bradykinesia, characteristic of PD, might lead to an underestimation of steps, especially when the activity monitor is worn on the wrist [33].

In our previous in-laboratory study involving 47 people with PD, we demonstrated a good criterion validity (intraclass correlation coefficient=0.76) and an acceptable level of error (mean absolute percentage error=5%) in step counting using a consumer smartwatch (Garmin Vivosmart 4), when worn on the side least affected by the disease and under well-controlled pharmacological conditions [31]. In the context of real-life settings, a separate study underscored the criterion validity of average daily steps (avDS) measured by several wrist-worn consumer devices (Fitbit Alta [Google] and Fitbit Inspire 3 [Google]) in 28 people with PD at home, compared with a research-grade device (Dynaport movemonitor) [34]. We recently replicated these results, using Garmin Vivosmart 4, in a proof-of-concept experiment [35]. Beyond criterion validity, the reliability of a measurement tool—the consistency of repeated measures in unchanged individuals—is equally crucial. In this regard, our recent findings indicate that a minimum of 4 days is necessary to obtain a reliable estimate of avDS recorded by a consumer smartwatch (Garmin Vivosmart 4) in free-living conditions [36].

An essential additional metrological characteristic of an activity monitor is its capability to reliably capture changes in measured parameters and the clinical significance of these changes. In this context, the minimum detectable change (MDC) and the minimal clinically important difference (MCID) are 2 key metrics and are pivotal for calculating clinical trial sample sizes and assessing the effectiveness of interventions [37,38]. On the one hand, MDC denotes the smallest change that can be confidently identified beyond the measurement error of the instrument used for a specific parameter [37]. In a recent work of ours on 56 individuals with mild-to-moderate PD, we found that for a monitoring period of at least 4 days, the MDC for the Garmin Vivosmart 4 ranged between 1198 and 1524 steps/day, averaging at 1366 steps/day [36]. On the other hand, MCID represents the smallest difference in a parameter that is meaningful from a clinical perspective [38]. In fact, a statistically significant change after an intervention may not represent a modification that has a real impact on the patient’s condition and, ultimately, might not be perceived by individuals. Therefore, a measure for clinical significance is required. MCID can be estimated in different ways, and there is no definite consensus for the best method [39,40]. Globally, two calculation methods exist: (1) distribution-based and (2) anchor-based. Distribution-based methods rely on the statistical characteristics of the variables of interest within the given population to identify the magnitude of change that would be unlikely to be observed by chance. Spread parameters, such as SD and SE of measurements, are commonly used indices to calculate MCID using distribution-based methods [39,40]. However, several authors underlined the limitation of distribution-based methods in capturing truly meaningful change and considered the use of these techniques only as an addition to anchor-based methods [39,41].

Anchor-based methods compare the change in patients’ variable of interest with how patients score on a second, explicit metric of improvement (ie, the anchor) [39,40,42]. Usually, when applying the anchor-based method, improvement is evaluated longitudinally following an intervention and a time period for which a change in the anchor measure is expected [39]. However, cross-sectional evaluation is also possible, as reported by Motl and collaborators [43]. With this second approach, a value, or a range of values, is defined for the anchor that corresponds to the MCID and then the target score of the variable of interest that corresponds to the anchor value is calculated. With the cross-sectional method, the between-subjects variability in the relationship between the anchor and the new measure is often high; therefore, multiple anchors are often needed to provide a well-triangulated MCID value [39]. However, this could offer the advantage of delineating several levels of MCID depending on their specific goals and expected outcomes, providing a more versatile framework [43]. While generally considered more reliable than distribution-based methods, potential limitations of anchor-based methods include recall bias and the need for at least a moderate correlation between the anchor and the variable of interest [39].

As of now, there are no published studies that have evaluated the MCID for avDS in people with PD. Therefore, considering these identified research gaps and the clinical significance of the psychometric and clinometric properties of daily steps measured by consumer smartwatches in free-living conditions for people with PD, the objective of this study was to determine the MCID of avDS as recorded by a consumer smartwatch in real-world settings for this population of individuals.

## Methods

### Population

Participants were recruited at the Movement Disorder Outpatient Service of the Sant’Andrea University Hospital, Rome, Italy, in the period between March 2023 and February 2024. Inclusion criteria were (1) diagnosis of idiopathic PD according to Movement Disorder Society criteria [44], (2) aged 18 years or older, (3) disease stage <4 according to the modified Hoeh and Yahr scale (mHY; “severe disability; still able to walk or stand unassisted”) [45], and (4) ability to perform the experimental procedure. Exclusion criteria were (1) cognitive impairment as defined by a Montreal Cognitive Assessment score < 21, and (2) orthopedic, rheumatologic, or systemic conditions affecting mobility as judged by the assessor.

### Ethical Considerations

This cross-sectional study was performed in accordance with the ethical standards as laid down in the 1964 Declaration of Helsinki and its later amendments. Approval was granted by the local Ethical Committee of Sapienza, University of Rome (Reference 0372/2022). Data collection and processing followed the current European regulation for data protection. All participants provided written informed consent (Multimedia Appendix 1) before the beginning of measurements. All data were deidentified. Participants did not receive any form of compensation.

### Demographic and Clinical Data

Participants were evaluated during scheduled visits. Demographics (age and sex) and anthropometric measures (weight, height, and BMI) were collected. Disease duration, disease stage according to the mHY scale, and levodopa equivalent daily dose (LEDD) [46] were also collected. Participants were then evaluated with the following rating scales:

The sum of the Movement Disorder Society Unified Parkinson’s Disease Rating Scale (MDS-UPDRS) [47] parts I and II was used to assess the impact of motor and nonmotor symptoms on daily life [48]. MDS-UPDRS part III was used to assess the severity of motor symptoms. From MDS-UPDRS part II and III scores, participants were classified into tremor dominant (TD), postural instability and gait disorder (PIGD), or indeterminate disease subtypes, according to Stebbins et al [49]. Participants were also grouped in those with mild and moderate disease severity based on the MDS-UPDRS score as proposed by Martínez-Martín and colleagues [50]. The Parkinson’s Disease Questionnaire-39 was used to evaluate participants’ quality of life [51]. The Short Physical Performance Battery (SPPB) was used to assess lower limb function and general physical capacity [52]. The Wearing-Off Questionnaire-19 was used to assess the presence of wearing off. A score ≥2 was used as a cutoff to define the presence of wearing off [53]. The Fatigue Severity Scale was used to assess fatigue severity; a score ≥4.67 was used to define clinically relevant fatigue [54]. The item 1 of the New Freezing of Gait Questionnaire [55] was used to assess the presence of FOG, defined as a score >0. Finally, the MDS-UPDRS item 2.10 was used to assess the absolute presence or absence of tremor as reported by participants.

### Experimental Procedure

Participants then received the smartwatch Garmin Vivosmart 4 to be worn at home for 5 consecutive days, including at least 1 weekend day, on the wrist of the body side least affected by the disease [31]. Participants were instructed to wear the smartwatch at all times during the day and night and remove it only when involved in water activities (eg, bathing, showering, and swimming). Participants were also asked to perform daily activities as usual. No additional reminder or instructions were provided to participants during the monitoring period. We chose a 5-day period since we demonstrated previously that a minimum of 4 days of monitoring is needed to reliably estimate daily step count in people with PD [36]. Each smartwatch was configured according to the producer’s recommendations by indicating the patient’s age, height, weight, and the wrist on which the smartwatch was worn (ie, left or right). During the monitoring period, participants could access information on the smartwatch screen, including the total number of steps, heart rate, and sleep time. The device did not provide instructions, alerts, or other messages to participants.

After 5 days, they returned the smartwatch to the hospital. From the smartwatch dashboard, the total daily number of steps for each day was recorded, and the avDS were calculated [36]. Compliance was assessed based on the participants’ dashboard data. We could assess that the device was worn through heart rate and activity data. We considered all recording days with ≥80% wear time while awake to be valid. We estimated the awake time as 24 hours minus the total sleep time as indicated by the device.

### Data and Statistical Analysis

The statistical analyses were performed using JASP v0.18.3.0 (JASP Team, University of Amsterdam), R v4.0.3 (R Core Team), and RStudio v2022.07.1+554 for Windows (R Foundation for Statistical Computing). Descriptive statistics were calculated for the examined variables. Normality of distributions was assessed by histogram and residual plots inspection.

To define MCID, we used an anchor-based method identifying three levels of variables, following the approach of Motl and collaborators [43]: (1) scales capturing subtle changes in global mobility and motor functions (ie, MDS-UPDRS part III and SPPB); (2) clinical and health-related measures including disease duration, mHY, LEDD, PD subtype, disease severity as assessed by MDS-UPDRS, age, sex, and BMI; and (3) disease-related patient-reported outcomes (PROs) including MDS-UPDRS parts I and II, the Parkinson’s disease questionnaire-39, presence of FOG, presence of wearing off, presence of fatigue, and presence of tremor.

Such an approach could provide ranges of MCIDs and options for MCID selection that are appropriate for specific populations and circumstances, including both assessor-based and PROs, in clinical trials and interventions involving people with PD [39].

Since previous studies have recommended a correlation coefficient of at least 0.3‐0.35 between the change score and the anchor [56], we first conducted a correlation analysis between avDS and the included variables using the Spearman test. Only associations with a correlation coefficient >0.3 were considered for MCID estimation.

We then conducted a linear regression between avDS and MDS-UPDRS part I+II and part III, mHY, SPPB, LEDD, and age for estimating the incremental change in daily steps per relevant variable change. To evaluate the relevant change, we used the already known MCID for each scale included in the regression analysis. Scales for which an MCID was not reported were excluded from the analysis. In case of asymmetric MCID (ie, different values for improvement and deterioration), we used the average value rounded to the higher next whole number.

For the remaining variables, we calculated mean and SE differences in avDS between groups by performing between-subjects ANOVA with Bonferroni-Holm post hoc pairwise comparisons in case of overall significance, when the variables included more than 2 classes. For 2-class variables, we used the Student *t* test for independent groups to compute the mean differences and SE in avDS. Cohen *d* was used to estimate the standardized effect size. MCID for the 3 levels was then calculated, and the average of the included variables and range was reported. The level of significance was set at α<.05.

## Results

### Overview

A total of 100 people with PD were enrolled in the study. All participants were monitored through Garmin Vivosmart 4 at home for a period of 5 consecutive days. No participants or days were excluded based on the prespecified compliance criteria. Participants took on average 5949 (SD 3034) daily steps, ranging from 357 to 12620. Details of demographic, anthropometric, and clinical variables in the enrolled population are illustrated in Table 1.

**Table 1.** Clinical, anthropometric, and demographic characteristics of enrolled population.

Characteristics		People with PD^a^ (n=100)
Age (y), mean (SD)		67.8 (8.5)
Height (cm), mean (SD)		171 (9.0)
Weight (kg), mean (SD)		76.0 (13.2)
BMI (kg/m^2^), mean (SD)		25.8 (3.5)
Sex, n (%)	
Male		67 (67)
Female		33 (33)
Disease duration (y), mean (SD)		6.2 (4.4)
LEDD^b^ (mg), mean (SD)		544 (298)
mHY^c^, median (IQR)		2 (2‐2.5)
MDS-UPDRS I+II^d^, median (IQR)		11 (6‐16)
MDS-UPDRS-III, median (IQR)		27 (21‐32)
PDQ-39^e^, mean (SD)		12.1 (9.4)
Disease subtypes, n (%)	
TD^f^		31 (31)
IND^g^		8 (8)
PIGD^h^		61 (61)
WO^i^		28 (28)
FOG^j^		13 (13)
Fatigue		23 (23)
Tremor		55 (55)
Disease severity, n (%)	
Mild		63 (63)
Moderate		37 (37)
SPPB^k^, median (IQR)		10 (9‐12)
avDS^l^, mean (SD)		5949 (3034)

a PD: Parkinson disease.

b LEDD: levodopa equivalent daily dose.

c mHY: modified Hoehn and Yahr scale.

d MDS-UPDRS: Movement Disorder Society Unified Parkinson Disease Rating Scale.

e PDQ-39: Parkinson’s disease questionnaire-39.

f TD: tremor dominant.

g IND: indeterminate.

h PIGD: postural instability and gait disorder.

i WO: wearing-off.

j FOG: freezing of gait.

k SPPB: Short Physical Performance Battery.

l avDS: average daily steps.

### MCID

#### Global Mobility and Motor Functions

**Figure 1.**
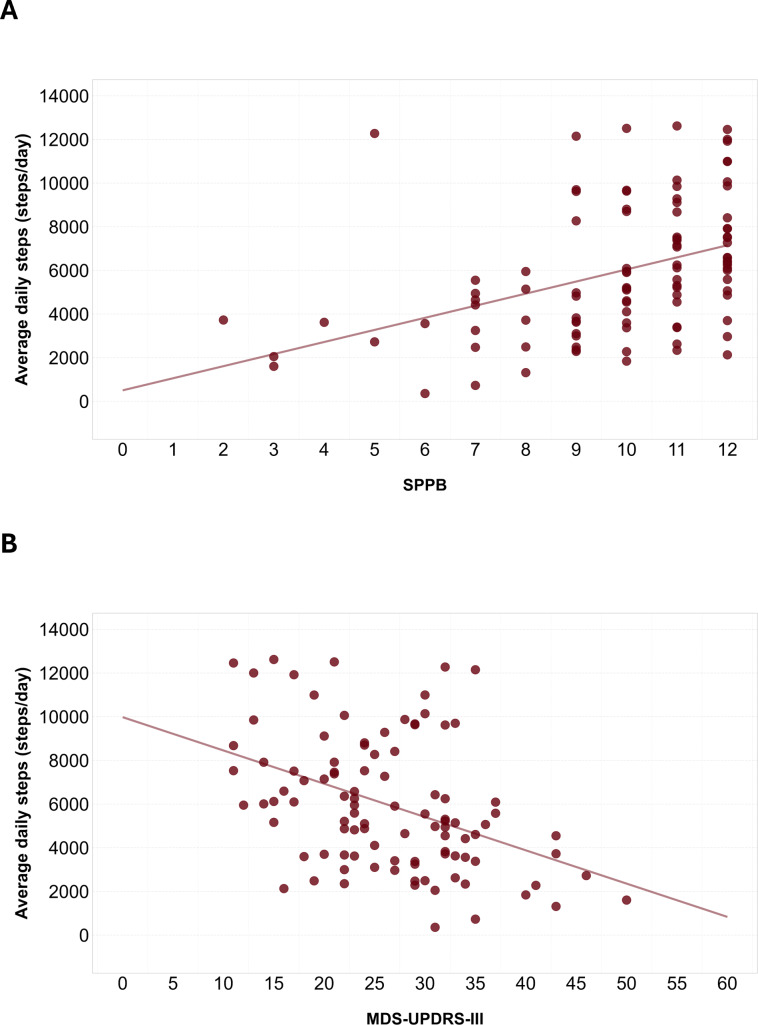
Correlation of average daily steps with Short Physical Performance Battery and Movement Disorder Society Unified Parkinson Disease Rating Scale-III. Scatterplot showing correlation between average daily steps and Short Physical Performance Battery score (panel A) and between average daily steps and Movement Disorder Society Unified Parkinson Disease Rating Scale-III score (panel B). A linear regression line was added to show the linear tendency. MDS-UPDRS: Movement Disorder Society Unified Parkinson Disease Rating Scale; SPPB: Short Physical Performance Battery.

All included variables showed a correlation coefficient >0.3 with avDS. The 2 linear regression models between avDS and MDS-UPDRS-III and SPPB were statistically significant (MDS-UPDRS-III: *F*_1,98_=20.46, *P*<.001, *R*^2^=0.16; SPPB: *F*_1,98_=19.84, *P*<.001; *R*^2^=0.16) (Figure 1).

The resulting regression equations indicated that every 1-point increase in MDS-UPDRS-III corresponded to a 152 steps/day decrease (avDS=9778−152×MDS-UPDRS-III). MCID for MDS-UPDRS was reported as 3.25 for score decrease and 4.63 for score increase [57]; therefore, we calculated a rounded average of 4 points, and we estimated a 608 steps/day modification for each 4-point change in MDS-UPDRS-III. Finally, each 1-point increase in SPPB score corresponded to an increase of 554 steps/day (avDS=503+554×SPPB). A change of 1 point is also the reported MCID for SPPB in older adults; therefore, we adopted this value [58]. Overall, the mean MCID across the 3 clinical scales that capture meaningful changes in motor and mobility condition was 581 steps/day (range 554‐608), corresponding to 10% of the avDS in our population.

#### Clinical and Health-Related Measures

**Figure 2.**
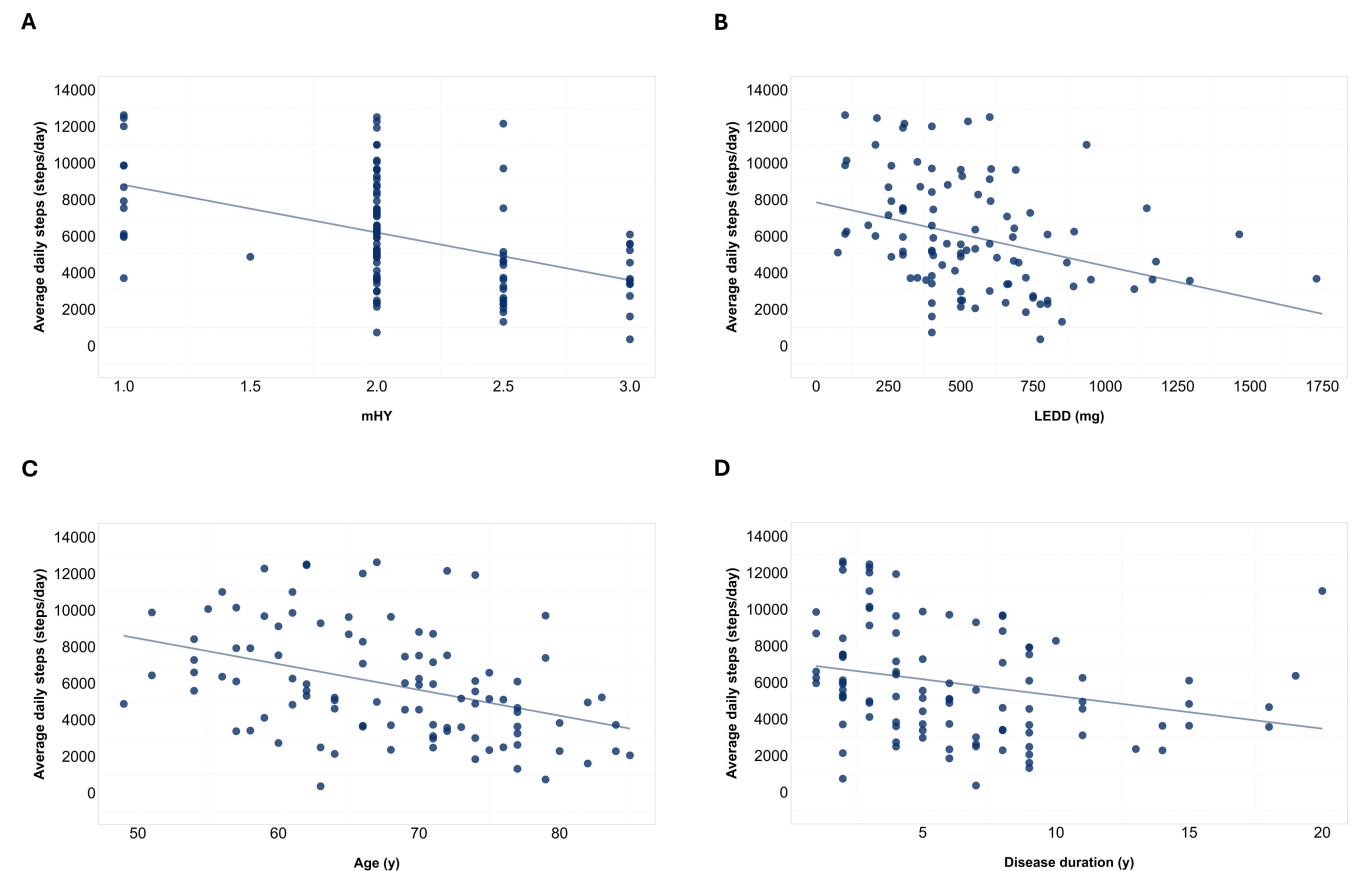
Correlation of average daily steps with modified Hoehn and Yahr stage, levodopa equivalent daily dose, age, and disease duration. Scatterplots are reported showing correlation between average daily steps and modified Hoehn and Yahr stage (panel A), levodopa equivalent daily dose (panel B), age (panel C), and disease duration (panel D). A linear regression line was added to show the linear tendency. LEDD: levodopa equivalent daily dose; mHY: modified Hoehn and Yahr stage.

All included variables except BMI showed a correlation coefficient >0.3 with avDS. Regression models of mHY, LEDD, age, and disease duration were statistically significant (mHY: *F*_1,98_=26.26, *P*<.001, *R*^2^=0.20; LEDD: *F*_1,98_=12.97, *P*<.001, *R*^2^=0.11; age: *F*_1,98_=18.22, *P*<.001, *R*^2^=0.15; disease duration: *F*_1,98_=7.01, *P*=.009, *R*^2^=0.06) (Figure 2).

The resulting regression equations indicated that every 1-point increase in mHY stage yielded a reduction of 2607 steps/day (avDS=11,409−2607×mHY). Since mHY included 0.5 steps increases between scores 1 and 3, we calculated that each step corresponded to a 1304 steps/day reduction.

For LEDD, regression equations indicated that every 1 equivalent mg increase led to a reduction of 3.5 steps/day (avDS=7846−3.5×mHY). Since the usual single dose of levodopa corresponds to 100 mg, we used this value as a reference, and we estimated a 350 steps/day modification for each 100 mg change in LEDD.

For age, regression equations indicated that a 1-year increase in age yielded a reduction of 141 steps/day (avDS=15,512−141×age). Furthermore, 10-year increases are usually used to divide participants in age groups; therefore, we estimated a 1410-step/day modification for each 10-year change of age.

For disease duration, regression equations indicated that a 1-year increase in disease duration resulted in a reduction of 180 steps/day (avDS=7071−180×age). We chose 5 years as a significant increment in disease duration since this was a common cutoff to categorize disease duration in previous studies [59-61]. Therefore, we estimated a 900-step/day modification for each 5-year change of disease duration.

**Figure 3.**
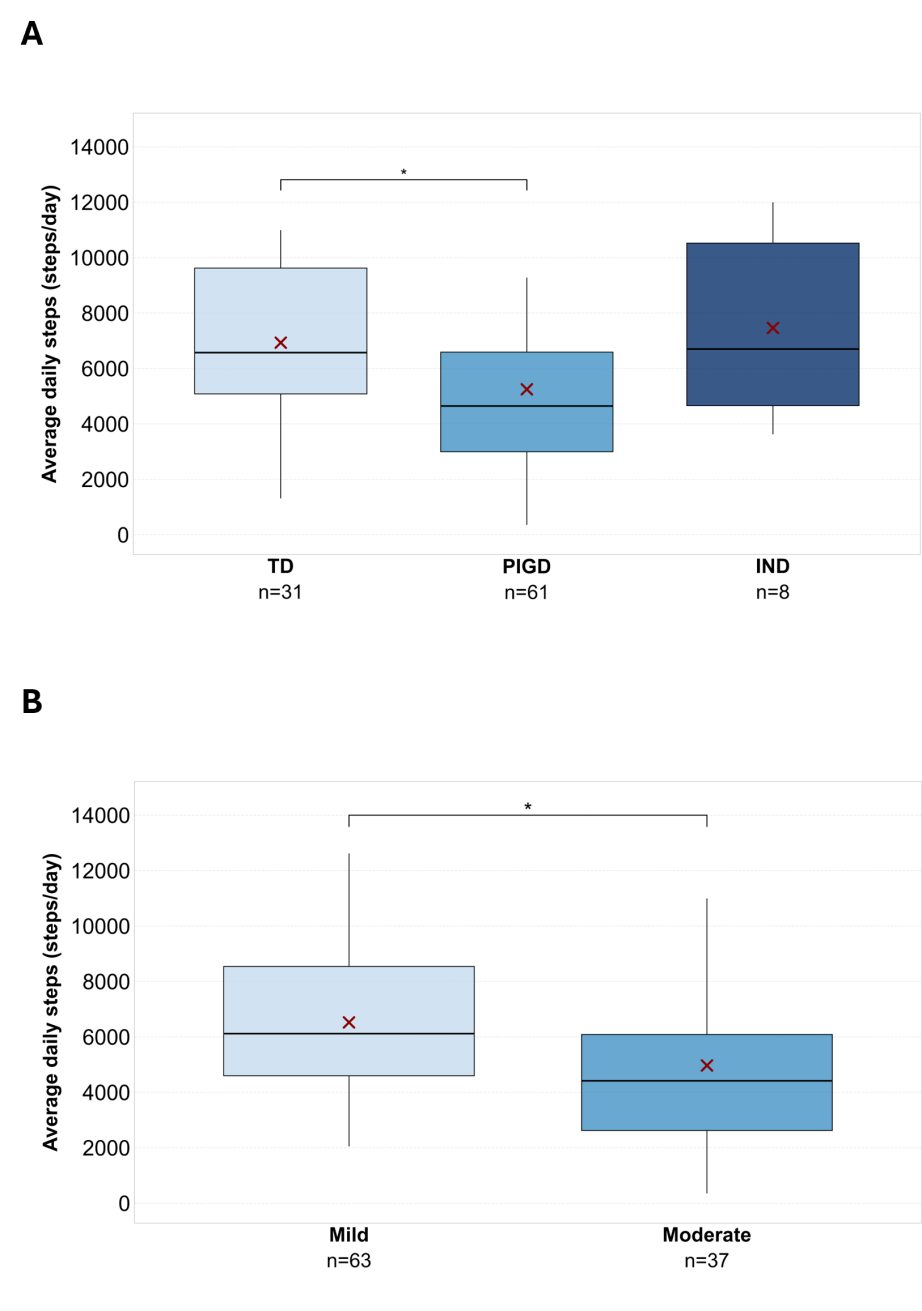
Differences across disease phenotypes, severity, and duration groups. Boxplots showing average daily steps in people with Parkinson disease across disease phenotypes (panel A) and disease severity (panel B). Thick line in the boxes indicates median; lower and upper box limits indicate first quartile (Q1) and third quartile (Q3), respectively; black vertical lines indicate lower and upper outliers boundaries calculated as Q1−(1.5×IQR) and Q3+(1.5×IQR), respectively. Red X indicates mean values for each disease group. Significant pairwise comparisons are marked by brackets and asterisks. IND: indeterminate; PIGD: postural instability and gait disorder; TD: tremor dominant. *P<.05.

Considering categorical descriptors, ANOVA showed a significant difference in avDS among participants with TD, PIGD, and indeterminate participants (*F*_2,97_=4.55, *P*=.01). Post hoc pairwise comparisons showed a significant difference between participants with TD and PIGD (*t*_90_=2.60, *P*=.03) with a mean difference of 1683 (SD 646) steps/day and a moderate effect size (Cohen *d*=0.57) (Figure 3A).

The Student *t* test showed a significant difference between mild and moderate disease severity, as categorized based on MDS-UPDRS cutoffs (*t*_98_=2.54, *P*=.01) with a mean difference of 1554 (SD 612) steps/day and moderate effect size (Cohen *d*=0.53) (Figure 3B).

No significant differences were found between males and females (*t*_98_=−0.12, *P*=.90).

Overall, the mean MCID across the clinical and health-related variables was 1200 steps/day (range 350‐1683), corresponding to 20% of the avDS in our population.

### Disease-Related PROs

**Figure 4.**
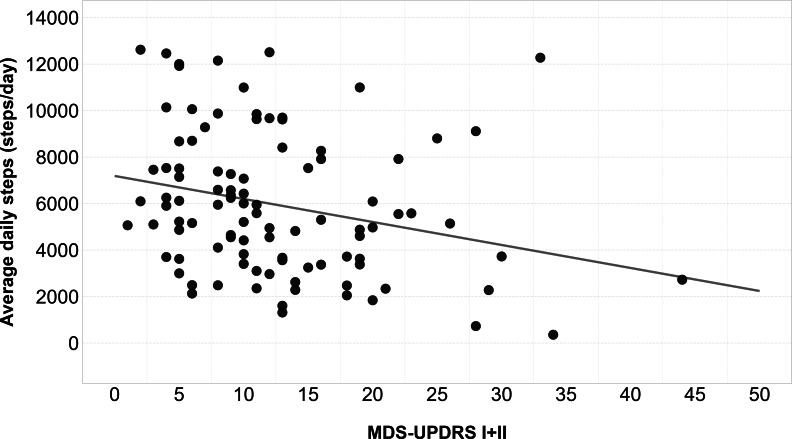
Correlation between average daily steps and Movement Disorder Society Unified Parkinson Disease Rating Scale I+II. A linear regression line was added to show the linear tendency. MDS-UPDRS: Movement Disorder Society Unified Parkinson Disease Rating Scale.

Only MDS-UPDRS I+II showed a correlation coefficient >0.3 with avDS and was included in the regression analysis. Regression models of MDS-UPDRS part I+II were statistically significant (MDS-UPDRS I+II: *F*_1,98_=7.03, *P*=.009, *R*^2^=−0.06) (Figure 4).

For MDS-UPDRS I+II, regression equations indicated that every 1-point increase led to a reduction of 99 steps/day (avDS=7188−99×MDS-UPDRS I+II). For the sum of MDS-UPDRS I and II, the reported MCID is 5.73 points for improvement and 4.70 points for worsening; therefore, we calculated a rounded average of 6 points, and we estimated a 594 steps/day modification for each 6-point change in MDS-UPDRS I+II.

**Figure 5.**
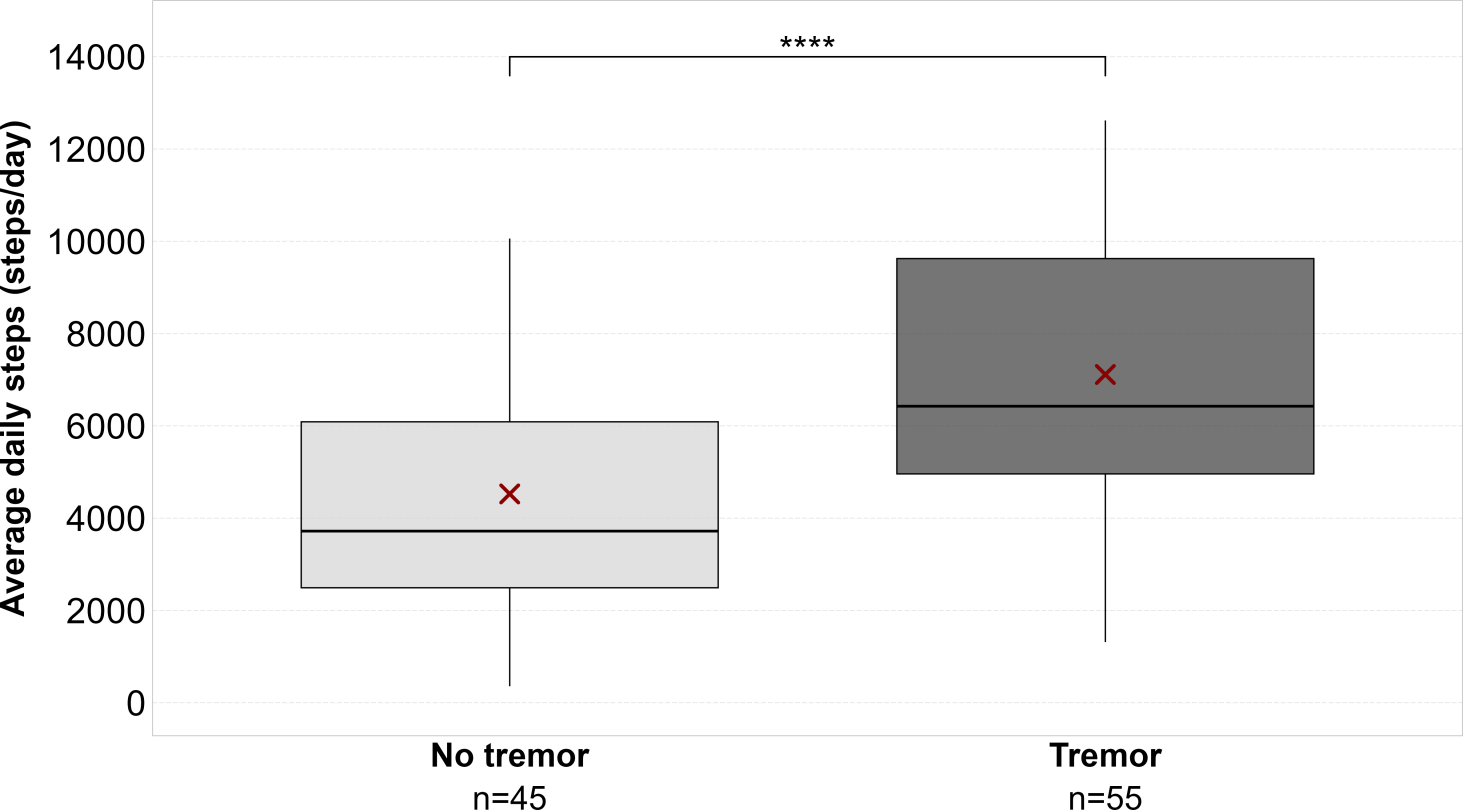
Differences between people with Parkinson disease with and without tremor. Boxplots are reported showing average daily steps in people with Parkinson disease with and without tremor. Thick line in the boxes indicates median; lower and upper box limits indicate first quartile (Q1) and third quartile (Q3), respectively; black vertical lines indicate lower and upper outliers boundaries calculated as Q1−(1.5×IQR) and Q3+(1.5×IQR), respectively. Red X indicates mean values for each disease group. Significant pairwise comparisons are marked by brackets and asterisks. PD: Parkinson disease. ****P<.0001.

The Student *t* test showed a significant difference between patients reporting tremor and those not reporting it (*t*_98_=−4.67, *P*<.001) with a mean difference of 2589 (SD 554) steps/day and a large effect size (Cohen *d*=−0.94) (Figure 5).

No significant differences were found in avDS between participants with and without FOG (*t*_98_=0.74, *P*=.46), between participants with and without fatigue (*t*_98_=0.61, *P*=.55), between participants with and without wearing-off (*t*_98_=−0.39, *P*=.70)

Overall, the mean MCID across the disease-related PROs variables was 1592 steps/day (range 594‐2589), corresponding to 27% of the avDS in our population.

The 3 calculated MCIDs, as well as the anchors and the avDS modification related to anchors significant changes, are reported in Table 2.

**Table 2.** Minimal clinically important differences for average daily steps for each of the 3 identified levels. The clinically significant anchor change and the corresponding average daily steps variation are reported next to each variable.

Anchors	avDS^a^ changes	MCIDs^b^
Global mobility and motor functions		581
MDS-UPDRS-III^c^ (4 points)	608	
SPPB^d^ (1 point)	554	
Clinical and health-related measures		1200
mHY^e^ (0.5 points)	1304	
LEDD^f^ (100 mg)	350	
Age (10 y)	1410	
Disease duration (5 y)	900	
Phenotype (TD^g^, PIGD^h^, or IND^i^)	1683	
Symptoms severity (mild or moderate)	1554	
Disease-related PROs^j^		1592
MDS-UPDRS I+II	594	
Tremor or no tremor (MDS-UPDRS 2.10)	2589	

a avDS: average daily steps.

b MCID: minimal clinically important difference.

c MDS-UPDRS: Movement Disorder Society Unified Parkinson Disease Rating Scale.

d SPPB: Short Physical Performance Battery.

e mHY: modified Hoehn and Yahr scale.

f LEDD: levodopa equivalent daily dose.

g TD: tremor dominant.

h PIGD: postural instability and gait disorder.

i IND: indeterminate.

j PRO: patient-reported outcome.

## Discussion

### Principal Findings

In this cross-sectional study, we aimed to calculate, for the first time, the MCID for avDS as an indicator of free-living walking behavior in people with PD using a consumer smartwatch under real-world conditions. We identified 3 distinct levels of MCID for avDS. The first level, of around 580 steps/day, is anchored to standardized measures of motor symptoms and mobility. The second level, of around 1200 steps/day, is based on clinical and health-related variables. Finally, the third level, of around 1590 steps/day, is anchored on disease-related PROs.

Globally, the MCID values ranged from 581 to 1592 steps/day, representing 10%‐27% of the average in our study population. We used the method used by Motl and colleagues [43], which offers the advantage of delineating distinct levels of clinically meaningful changes in real-world walking behavior for people with PD. These 3 thresholds provide a versatile framework for assessing the impact of clinical interventions in people with PD, depending on their specific goals and expected outcomes. Furthermore, the MCID estimates for avDS can be used either independently or in conjunction with other MCIDs, as presented in this manuscript for clinical assessment tools, to offer a more comprehensive evaluation of clinically meaningful changes in individuals with PD.

Our results suggest that a value of around 580 steps/day might reflect the smallest clinically meaningful change in avDS when measured by a consumer smartwatch and should be considered when designing a study including interventions focused on mobility or motor condition improvement. Differently, when global disease and health status are the main outcomes, our results showed that a larger value of around 1200 steps/day might be considered to define a clinically meaningful change.

In the last decades, a growing importance has been attributed to PROs, such as patient-reported scales, quality of life questionnaires, etc, as vital indicators of people’s perception of their own condition [62]. PROs are often central outcomes in patients’ perspectives and might capture some aspects that cannot be evaluated through objective measurements. The use of these outcomes is encouraged to guide research and clinical practice in the context of a patient-centered model of care, and several PROs were included in a recent consensus on outcome measures for PD [63]. To this end, in this paper, we calculated that a change of around 1590 steps/day might be considered to define a clinically meaningful intervention when patients’ self-perception is the main outcome.

It is also vital to consider the feasibility of achieving changes in avDS of the magnitudes identified in this study. Previous research has explored interventions in people with PD that use daily steps as an outcome measure [17,64]. In 1 study by Handlery and colleagues [17], people with PD who initially took fewer than 4200 steps per day managed to increase their daily step count by approximately 1250 steps/day 6 months after participating in high-intensity treadmill exercises [17]. Similarly, another randomized controlled trial by Ellis and colleagues [64] involving 44 people with PD found that participants who initially averaged fewer than 7500 steps per day increased their avDS by about 763 steps/day over a year, following a mobile health–supported exercise program. These findings suggest that achieving changes comparable with the MCID values identified in our study is feasible through interventions aimed at promoting physical activity.

Nevertheless, the capability of consumer devices to accurately detect changes of the magnitude reported here is crucial for their clinical utility. In a recent study of ours, we found that for a monitoring duration of at least 4 days, the MDC for the Garmin Vivosmart 4 ranged between 1198 and 1524 steps/day, averaging at 1366 steps/day [36]. This MDC value is higher than the smaller MCID we calculated, indicating that any observed change exceeding this threshold is not only beyond the realm of measurement error but also clinically significant. However, this also implies that the device might not be sensitive enough to consistently detect subtler yet clinically meaningful changes in avDS, as indicated by the lower MCID limit of approximately 580 steps/day. Consequently, while the Garmin Vivosmart 4 can be effective in measuring significant differences in avDS in people with PD, for smaller anticipated changes, other instruments with higher reliability need to be considered. Future research should therefore investigate the performance of various consumer smartwatches and other devices such as smart socks, insoles, etc, and their applicability across different neurological conditions to validate, refine, and possibly extend our findings.

### Limitations

We recognize certain limitations in our study. First, the people with PD included in our study displayed relatively high levels of physical capacity, as evidenced by a median SPPB score of 10 (IQR 9-12), and adequate cognitive functions, due to our exclusion of participants with a Montreal Cognitive Assessment score below 21. In addition, those with more advanced disease stages or requiring walking aids were not part of our study group. This selection criterion potentially limits the generalizability of our findings to only mild-to-moderate people with PD. Indeed, in a population of individuals with worse cognitive and motor conditions, we might expect lower accuracy and reliability of avDS estimates by smartwatches and fewer steps/day due to greater walking impairments [65-67]. Therefore, higher absolute and relative MCID values as well as different anchor combinations could be observed. Nevertheless, the sample in our study can be seen as representative of the typical target demographic for interventions using consumer-grade wearable technology. In fact, including people with PD with a disease stage greater than 3, those using walking aids, or with more severe cognitive impairments poses significant challenges in the use of consumer technology and was beyond our study’s scope. Eventually, the evaluation of people using walking aids with commercial out-of-the-shelf devices, without more advanced data processing, might not even be feasible due to the potential inadequate accuracy of a smartwatch for measuring daily steps among this patient population, whose mobility characteristics differ substantially from those of individuals with physiological ambulation [68]. Future research incorporating participants with PD with lower functional scores, increased disease severity, and more pronounced cognitive impairments would be valuable.

In addition, while we incorporated a range of general and disease-specific measurements, other factors that might affect daily life mobility, such as fear of falling, frontal executive cognitive dysfunction, and objectively measured gait parameters (eg, gait speed, cadence, and step length), were not examined. Furthermore, specific subdomains of adopted measurement tools could be further explored (eg, walking speed or balance subitems of SPPB and PIGD subscore of MDS-UPDRS part III). Further studies exploring the relationship between these factors and smartwatch-recordable parameters are recommended to provide a more comprehensive understanding of mobility in people with PD.

In addition, we did not extract the raw data from the smartwatches, but used the information reported by the device’s dashboard. While acknowledging the technical limitations of this approach, we designed the study to mimic as closely as possible the actual use of the device in a real-world context and to use parameters as similar as possible to those that a general user would have access to. Finally, in this work, we used an anchor-based cross-sectional method to define different levels of MCID as presented by Motl et al [43]. However, other anchor-based methods are available. In particular, a widely used method consists of the longitudinal calculation of MCID following an intervention [39,40]. However, while this latter method could allow for drawing more sound conclusions, it must be considered that it might be dependent on the type of interventions used, thus hampering the generalizability. Furthermore, previous evidence highlighted the sample-specificity of MCID estimates (particularly for distribution-based methods) [69] and the dependency on the baseline scores of the enrolled population [70]. This further stresses the importance of conducting future longitudinal, multicenter studies in a free-living context, including independent cohorts of people with PD with different clinical baseline conditions, from different clinical centers—and even different countries—and using different evaluation tools to confirm and expand the results hereby presented. In addition, since different methods to calculate MCID could lead to different results [71], using different techniques and comparing them will be important to provide the best scientific evidence on this topic. Nevertheless, we considered it relevant to provide researchers with a first indication of MCID for avDS as measured by a consumer smartwatch in order to build a framework to interpret findings in studies incorporating this outcome measure. In fact, researchers designing a study (eg, a clinical trial that aims at assessing the effectiveness of an intervention specifically designed to increase physical activity in PD) and incorporating smartwatch-based avDS as an outcome measure of physical activity will be able to define end points that are clinically relevant, beyond statistical significance. Furthermore, based on the other included endpoints (ie, mobility, clinical indicators, or PROs), investigators will be able to select the more appropriate MCID level.

### Conclusions

We reported 3 levels of MCID for avDS measured through Garmin Vivosmart 4, for 5 consecutive days, 1 of approximately 580 steps/day (or about 10% of our population’s average), anchored to standardized measures of motor symptoms and mobility. A second, of around 1200 steps/day (or about 20% of the mean in our population), anchored to clinical and health-related variables. Finally, a third, or around 1590 steps/day (or about 27% of the mean in our population) anchored to disease-related PROs. Considering that the previously reported MCD for Garmin Vivosmart 4 was 1366 steps/day, this indicates that any observed change exceeding this threshold is not only beyond the realm of measurement error but also clinically meaningful. Furthermore, based on previous studies, we could suppose that achieving changes comparable with the smaller MCID identified in our study might be feasible following interventions aimed at promoting physical activity in people with PD. These findings, together with existing evidence for reliability and validity on consumer smartwatches, could be relevant for designing future clinical trials involving avDS as an outcome measure of real-world walking behavior in PD, and evaluating the effectiveness of intervention promoting free-living walking.

## Supplementary material

10.2196/64213 Multimedia Appendix 1 Informed consent form.

## Abbreviations

AvDS: average daily steps
FOG: freezing of gait
LEDD: levodopa equivalent daily dose
MCID: minimal clinically important difference
MDC: minimum detectable change
MDS-UPDRS: Movement Disorder Society Unified Parkinson’s Disease Rating Scale
mHY: modified Hoeh and Yahr scale
PD: Parkinson disease
PIGD: postural instability and gait disorder
PRO: patient-reported outcome
SPPB: Short Physical Performance Battery
TD: tremor dominant
