# Supplementary material for: Minimal Clinically Important Difference of Average Daily Steps Measured Through a Consumer Smartwatch in People With Mild-to-Moderate Parkinson Disease: Cross-Sectional Study
Source: JMIR Mhealth Uhealth. 2025 Jul 29;13:e64213. doi: 10.2196/64213 (PMC12306920; doi:10.2196/64213)
Supplement: Multimedia Appendix 1 [file mhealth-v13-e64213-s001.pdf]

UMBERTO I
POLICLINICO DI ROMA
DAI NEUROLOGIA E PSICHIATRIA

Versione 1 del Protocollo Sperimentale Roma, 08/03/2022

MODULO DI CONSENSO INFORMATO

Titolo dello Studio: "Valutazione strumentale di sintomi motori mediante utilizzo di sensori indossabili in pazienti affetti da Malattia di Parkinson". Protocollo n.

Promotore: Dipartimento Neuroscienze Umane

1	Confermo di aver letto e compreso quanto esposto nel foglio informativo (versione 1 del 08/03/2022) ed aver avuto l'opportunità di porre domande a cui ho ricevuto delle risposte soddisfacenti e che ho avuto abbastanza tempo per prendere la mia decisione.	☐
2	Ho compreso che la mia partecipazione è volontaria e che sono libero di ritirarmi in qualsiasi momento, senza dare alcuna spiegazione e che, se lo farò, ciò non pregiudicherà la cura e le attenzioni future da parte del medico.	☐
3	Sono consapevole che i miei dati clinici possono essere visionati da personale autorizzato così come dagli Enti Regolatori. Acconsento affinché queste persone possano accedere ai miei dati che sono rilevanti per questo studio.	☐
4	Confermo di voler partecipare allo studio proposto.	☐
5	Confermo di ricevere copia del foglio informativo e copia di questo modulo di consenso informato debitamente firmato.	☐

Nome e cognome del soggetto (se abile)	Firma	Data
Nome e cognome del Rappresentante Legale (se applicabile)	Firma	Data
Nome e Cognome del testimone imparziale (se applicabile)	Firma	Data
Nome e cognome di chi somministra il consenso	Firma	Data

DAI NEUROSCIENZE UMANE
Viale dell'Università 30 - 00185 Roma
Indirizzo web: www.neurosapienza.it
Centralino Neuroscienze Umane: 0649914762
Ambulatorio Neurologia: 0649914493 10.00-12.00
Centralino Azienda Policlinico Umberto I : 0649971
